# In Silico Drug Repurposing Uncovered the Antiviral Potential of the Antiparasitic Drug Oxibendazole Against the Chikungunya Virus

**DOI:** 10.1021/acsomega.4c03417

**Published:** 2024-06-13

**Authors:** Vitor W. Rabelo, Maria Leonisa Sanchez-Nuñez, Leonardo S. Corrêa-Amorim, Richard J. Kuhn, Paula A. Abreu, Izabel C. N. P. Paixão

**Affiliations:** † Programa de Pós-graduação em Ciências e Biotecnologia, Instituto de Biologia, Universidade Federal Fluminense, Niterói, Rio de Janeiro CEP 24210-201, Brazil; ‡ Gerência de Desenvolvimento Tecnológico, Instituto Vital Brazil, Niterói, Rio de Janeiro 24230-410, Brazil; § Department of Biological Sciences, Purdue University, West Lafayette, Indiana 47907, United States; ∥ Purdue Institute of Inflammation, Immunology, and Infectious Disease, Purdue University, West Lafayette, Indiana 47907, United States; ⊥ Instituto de Biodiversidade e Sustentabilidade (NUPEM), Campus Macaé, Universidade Federal do Rio de Janeiro, Rio de Janeiro CEP 27965-045, Brazil; # Departamento de Biologia Celular e Molecular, Instituto de Biologia, Universidade Federal Fluminense, Niterói, Rio de Janeiro CEP 24210-201, Brazil

## Abstract

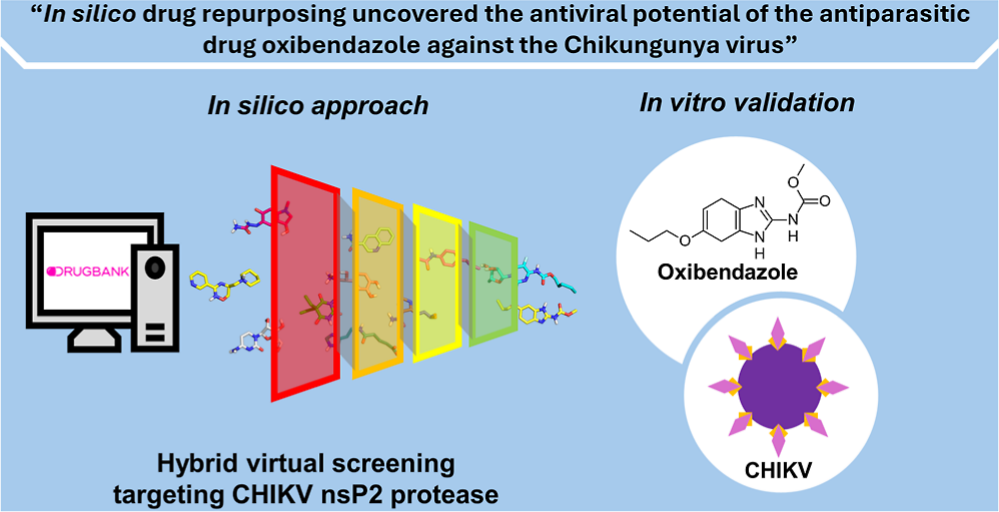

Chikungunya virus (CHIKV) has been reported in over 120 countries and is the causative agent of Chikungunya fever. The debilitating nature of this disease, which can persist months to years after acute infection, drastically impacts the quality of life of patients. Yet, specific antivirals are lacking for the treatment of this disease, which makes the search for new drugs necessary. In this context, the nsP2 protease emerges as an attractive therapeutic target, and drug repurposing strategies have proven to be valuable. Therefore, we combined in silico and in vitro methods to identify known drugs as potential CHIKV nsP2 protease inhibitors with antiviral properties within DrugBank. Herein, we developed a hybrid virtual screening pipeline comprising pharmacophore- and target-based screening, drug-like, and pharmaceutical filtering steps. Six virtual hits were obtained, and two of them, capecitabine (CPB) and oxibendazole (OBZ), were evaluated against CHIKV replication in Vero cells. CPB did not present antiviral activity, whereas OBZ inhibited the replication of two different strains of CHIKV, namely, 181-25 (Asian genotype) and BRA/RJ/18 (clinical isolate from ECSA genotype). OBZ showed potent antiviral activity against the CHIKV BRA/RJ/18 (EC_50_ = 11.4 μM) with a high selectivity index (>44). Analogs of OBZ (albendazole, fenbendazole, and mebendazole) were also evaluated, but none exhibited anti-CHIKV activity, and further, their stereoelectronic features were analyzed. Additionally, we observed that OBZ acts mainly at post-entry steps. Hence, our results support further in vivo studies to investigate the antiviral potential of OBZ, which offers a new alternative to fight CHIKV infections.

## 1 Introduction

Chikungunya virus (CHIKV) is an enveloped positive-sense single-stranded RNA virus that belongs to the Togaviridae family and *Alphavirus* genus. The virus genome has a size of approximately 11.8 kb and contains two open reading frames (ORF): the 5′-ORF encodes four nonstructural proteins (nsP1–4), while the 3′-ORF encodes six structural proteins or peptides, such as capsid (C), envelope proteins 1, 2, and 3 (E1, E2, and E3), 6K, and transframe (TF).^1^ According to its genetic features and geographic distribution, this virus is classified into three major genotypes, namely, Asian, West African, and East-Central-South-African (ECSA), with the Asian and ECSA ones being the most predominant worldwide.^2^ CHIKV is the etiological agent of Chikungunya fever and is primarily transmitted by *Aedes* spp. mosquitoes, especially *Ae. aegypti* and *Ae. albopictus*.^3^

Chikungunya fever has been recognized as a neglected disease and has been reported in over 120 countries from all continents, except for Antarctica.^4,5^ In the last two decades, over 10 million cases have been notified globally and, since 2016, Brazil has been the epicenter of the disease’s epidemics in Latin America with over 1.6 million cases.^6,7^ Currently, Brazil still holds the highest number of cases worldwide, followed by Paraguay, Argentina, and Bolivia.^8^ In 2023, many Latin American countries experienced a remarkable increase in CHIKV cases and deaths, which led the World Health Organization to issue an alert for enhancing surveillance and control strategies in the region.^9^ In addition, climate changes along with globalization significantly affect mosquitoes distribution^10^ and approximately 50% of the global population will likely be at risk for arbovirus transmission in the next decades.^11^

Usually, Chikungunya fever is self-limited and characterized by fever, rash, arthralgia or polyarthralgia, myalgia, headache, and edema.^12^ However, nearly 40% of patients evolve into the chronic phase and experience debilitating arthralgia or polyarthritis that persists for months to years after acute infection.^13^ Other severe complications can also occur, such as Guillain–Barré syndrome, encephalitis, retinitis, stroke, pneumonia, and liver and kidney failure.^12,14^ The economic burden of this disease due to its debilitating nature and costs associated with hospitalization and diagnostics is also raising concerns in many countries.^15^ Recently, the FDA has approved a CHIKV vaccine,^16^ but there is still a lack of specific antivirals for the treatment of this disease, which highlights the need for new drugs.^17^

Drug repurposing is a promising strategy for identifying new medical applications for approved and investigational drugs. This approach offers several advantages, such as reduced time and costs of the drug development process, because pharmacokinetic and safety profiles and formulation studies have been extensively investigated before.^18^ In the context of CHIKV drug discovery, the CHIKV nsP2 protease emerges as a valuable therapeutic target since this enzyme is critical for CHIKV replication and pathogenesis. The nsP2 protease is a cysteine-protease responsible for nonstructural polyprotein processing and maturation of nonstructural proteins^19^ and is involved in the host cell shutoff.^20^ Previously, our group investigated the structural basis underlying the inhibition of this enzyme by using computer-aided techniques,^21^ which prompted us to apply this knowledge in the search for new inhibitors within DrugBank libraries. In this work, we carried out a hybrid virtual screening within DrugBank to search for potential nsP2 protease inhibitors and further evaluated their in vitro antiviral properties as well as their mechanism of action to validate our in silico studies and identify new drug repurposing opportunities to fight CHIKV infections.

## 2 Results

### 2.1 Virtual Screening of Potential CHIKV nsP2 Protease Inhibitors within DrugBank Libraries

**Figure 1:**
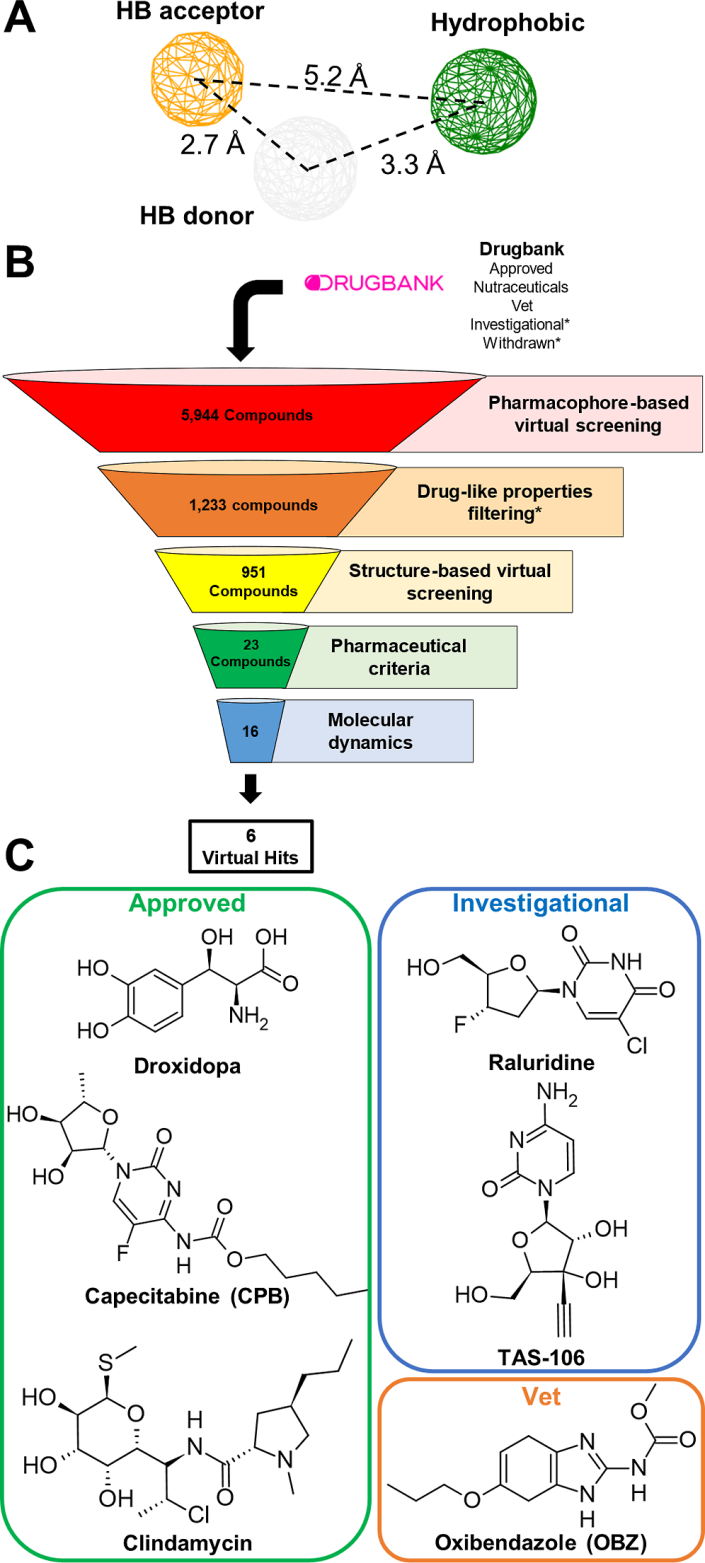
Virtual screening results for the discovery of potential CHIKV nsP2 protease inhibitors within the DrugBank libraries (approved, nutraceuticals, vet, withdrawn, and investigational). (A) Pharmacophore model (model F); (B) overall workflow for the virtual screening. *Compounds obtained from withdrawn, and investigational libraries were submitted to the drug-like filtering step. (C) 2D structures of the virtual hits obtained.

Previously, our group investigated the binding mode and dynamics behavior of CHIKV nsP2 protease inhibitors using in silico simulations.^21^ Based on our findings, we constructed six pharmacophore models (Figure S1) to screen DrugBank libraries to identify known drugs with anti-CHIKV activity. First, we assessed whether these models could recover known nsP2 protease inhibitors from our test set. Among models, model F was the only one able to recover all inhibitors (Table S1) and, consequently, was employed for the pharmacophore-based virtual screening. Model F was constructed based on the binding mode of structurally similar inhibitors **C5**, (*SS*)-**C14**, and (*S*)-**C16** and comprises three pharmacophores (Figure 1A): (i) a hydrogen-bond acceptor group to interact with the catalytic cysteine residue (C1013); (ii) a hydrogen-bond donor group, to interact with N1082; and (iii) a hydrophobic group to establish interactions with Y1079, N1082, H1083, or W1084.

After screening 5944 compounds from different libraries, 1233 ligands were selected based on model F. Following this, duplicates and compounds with undesirable drug-like properties, particularly the ones from withdrawn and investigational libraries, were removed, yielding 951 ligands, which were further submitted to the target-based virtual screening (Figure 1B).

Before conducting the target-based steps, we evaluated the ability of 10 scoring functions to discriminate true inhibitors from decoys using the ROC curve (Table S2). Only the ad4_scoring function yielded an AUC-ROC value ≥ 0.70. We then assessed a consensus scoring strategy by calculating the average *Z*-score of the combined scores obtained from the scoring functions with AUC-ROC ≥ 0.60. Among the tested combinations, the consensus results from the smina, vinardo, and ad4_scoring functions resulted in the highest AUC-ROC value (0.72). Hence, this methodology was employed for reranking ligands after docking studies, and 20% of the top-scoring ligands were visually inspected. A total of 23 ligands were selected at this step, and 7 were removed based on pharmaceutical criteria, such as severe side effects (e.g., neuropathy) and limited administration routes (e.g., topical only). Finally, the 16 ligands in complex with nsP2 protease were submitted to molecular dynamics (MD) simulations for 30 ns and 6 ligands were selected as virtual hits (Figure 1B).

**Figure 2:**
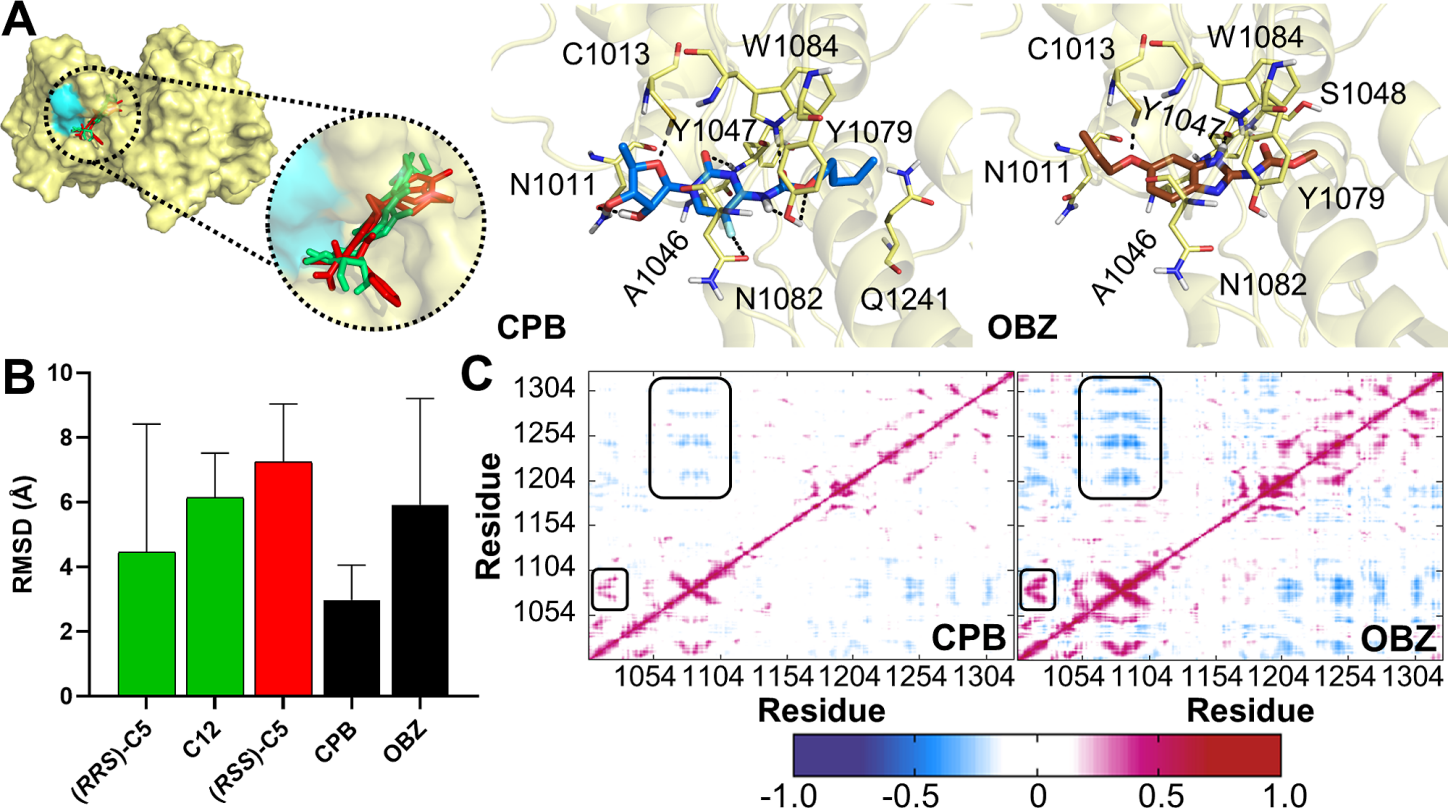
Molecular modeling results of the virtual hits obtained from virtual screening and purchased for experimental validation. (A) Molecular docking of these two drugs with the nsP2 protease (PDB code 3TRK) and their interactions. In the first panel, superimposition of the binding mode of the virtual hits (green) with known inhibitors, (*RRS*)-**C5** and **C12** (red), is shown. The surface of the catalytic dyad (C1013 and H1083) is shown in cyan. Hydrogen bonds are shown as dashed lines. In addition, molecular dynamics results are shown for these two ligands: (B) average ligands’ RMSD values (±SD) along the 30 ns simulation. For comparison, we included the values obtained for known inhibitors, (*RRS*)-**C5** and **C12**, and an inactive ligand, ((*RSS*)-**C5**); (C) dynamic cross-correlation matrix (DCCM) analysis for the protein in complex with the two ligands. Positive values close to 1.0 (red) indicate directly correlated movements, while negative values close to −1.0 (blue) indicate inversely correlated movements. Conserved movements of the protein complexed with the known inhibitors (*RRS*)-**C5** and **C12** are detached.

The virtual hits belong to different DrugBank libraries, such as human (e.g., droxidopa, capecitabine, and clindamycin) or vet (e.g., oxibendazole)-approved drugs and investigational ones (e.g., raluridine and TAS-106) (Figure 1C). Among them, two drugs, capecitabine (CPB; anticancer) and oxibendazole (OBZ; antiparasitic), were selected and purchased for experimental validation. Thereafter, we briefly presented the molecular modeling results focused on these two drugs (Figure 2).

In molecular docking studies, OBZ and CPB were superimposed to the known inhibitors (*RRS*)-**C5** and **C12** and interacted with, at least, one catalytic residue (Figure 2A). Both compounds were hydrogen-bonded to the catalytic C1013 as well as with Y1047 and Y1079, while a van der Waals interaction was maintained with A1046. They also shared interactions with several residues, such as N1011, N1082, and W1084, though they presented different natures. For instance, OBZ interacted with these residues by van der Waals contacts, while CPB was involved in hydrogen bond interactions with them. In addition, CPB and OBZ interacted with Q1241 and S1048, respectively, through van der Waals interactions.

In the MD simulation, we used previously defined criteria for selecting the hits, such as the ligands’ fluctuation measured by its RMSD (Figure 2B). CPB showed less fluctuation changes than the known inhibitors (*RRS*)-**C5** and **C12**, with average RMSD values of 2.98, 4.46, and 6.14 Å, respectively. OBZ had increased fluctuations and exhibited an RMSD of 5.92 Å, which still indicated higher stability within the enzyme’s active site compared to **C12** and the inactive ligand (*RRS*)-**C5** (7.25 Å). Another criterion used was the ligands’ effects on protein movements, which was analyzed by the dynamic cross-correlation matrix (DCCM). This analysis showed that CPB and OBZ had similar effects as known inhibitors (*RRS*)-**C5** and **C12** with predominant inversely correlated movements, whereas the correlated movements of the catalytic dyad (C1013 and H1083) were maintained (Figure 2C). Therefore, these results indicated that virtual hits have structural features comparable to active ligands that are required to inhibit CHIKV nsP2 protease and support further in vitro validation assays.

### 2.2 Antiviral and Cytotoxicity Evaluation of the Virtual Hits OBZ and CPB Against CHIKV

The antiviral effect of the two virtual hits, OBZ and CPB, was evaluated against the replication of the CHIKV 181–25 strain (Asian genotype) in Vero cells. At 50 μM, CPD did not inhibit CHIKV replication, but OBZ significantly reduced virus titer by 66.4%, like the positive control CQ (98.2%) (Table 1). To exclude OBZ-mediated toxicity, we assessed the cytotoxic profile of this drug by MTT assay. OBZ did not show significant toxicity, and its CC_50_ exceeded the highest concentration tested (500 μM), whereas CQ presented higher cytotoxicity in Vero cells, with a CC_50_ value of 469.1 μM (Table 1 and Figure S2).

**Table 1:** Cytotoxicity and Antiviral Activity of Oxibendazole (OBZ), Capecitabine (CPB), and the Control Drug Chloroquine (CQ) Against the Replication of CHIKV 181-25 and BRA/RJ/18 Strains in Vero Cells

compound	CC_50_ (μM)	CHIKV 181-25	CHIKV BRA/RJ/18		
		inhibition (%)^a^	inhibition (%)^a^	EC_50_ (μM)	SI^b^
**CPB**	ND	0 ± 0	ND	ND	ND
**OBZ**	>500	66.4 ± 2.5	61.8 ± 2.7	11.4 ± 3.2	>44.0
**CQ**	469.1 ± 84.4	98.2 ± 0.2	48.5 ± 11.7	47.5 ± 10.2	9.9

a Inhibition rate of CHIKV replication at 50 μM (% relative to control). ND: not determined.

b Selectivity index (SI) was calculated as the ratio between CC_50_ and EC_50_ values.

Moreover, the antiviral effect of OBZ (50 μM) was assessed against the replication of a more virulent CHIKV strain, which is a Brazilian CHIKV isolate (BRA/RJ/18) that belongs to the ECSA genotype (Table 1). Interestingly, OBZ maintained its antiviral activity with an inhibition rate of 61.8%, while CQ had a lower activity (48.5%). Further, these compounds were evaluated at different concentrations to determine their antiviral potency against this strain (Table 1 and Figure S2). OBZ showed a nearly 4-fold increased potency in comparison to CQ with EC_50_ values of 11.4 and 47.5 μM, respectively. Besides being more active, OBZ showed remarkably higher selectivity (SI > 44.0) than CQ (SI = 9.9).

### 2.3 Evaluation of the Cytotoxicity, Antiviral Activity, and Stereoelectronic Properties of Marketed OBZ Analogs

**Figure 3:**
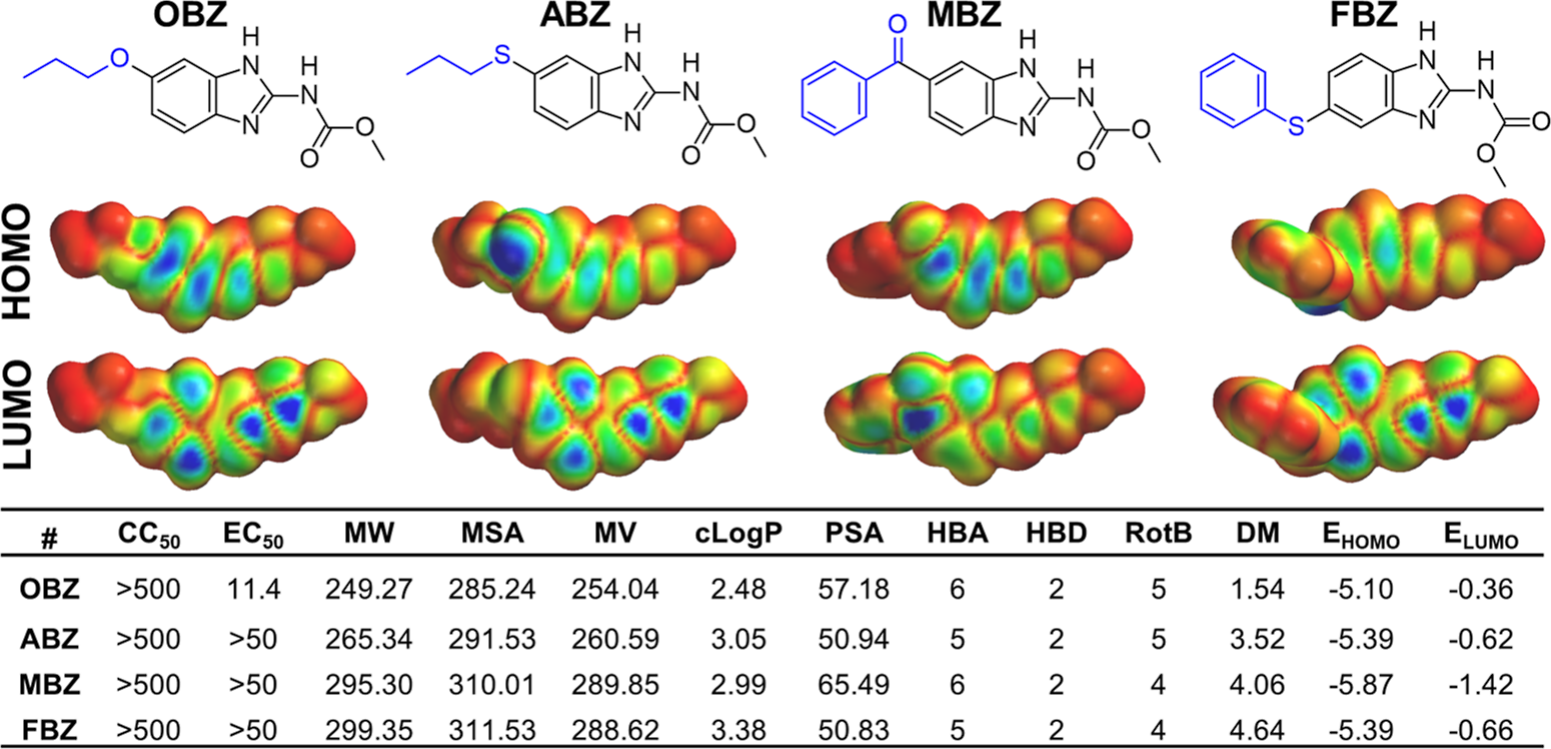
Cytotoxicity (CC_50_, μM), anti-CHIKV activity against BRA/RJ/18 strain replication in Vero cells (EC_50_, μM), and calculated stereoelectronic descriptors and maps of oxibendazole (OBZ) and its analogs albendazole (ABZ), mebendazole (MBZ), and fenbendazole (FBZ). Molecular weight (MW) (Da), molecular surface area (MSA) (Å^2^), molecular volume (MV) (Å^3^), calculated *n*-octanol/water partition coefficient (cLogP), polar surface area (PSA) (Å^2^), number of hydrogen-bond acceptors and donor groups (HBA and HBD, respectively), number of rotatable bonds (RotB), dipole moment (DM) (Debye), and HOMO and LUMO energies (*E*_HOMO_, *E*_LUMO_, eV). HOMO and LUMO density maps are encoded onto a van der Waals surface (0.002 e/au^3^) with density coefficient ranging from 0 (deepest red) to 0.018 (deepest blue).

OBZ is an approved drug for treating animal parasitic infections, like its analog fenbendazole (FBZ), while other analogs, such as albendazole (ABZ) and mebendazole (MBZ), are approved for human use. Thus, we evaluated the cytotoxic and antiviral profile of these analogs toward the CHIKV BRA/RJ/18 replication in Vero cells. The three analogs showed low cytotoxicity with CC_50_ values higher than 500 μM, but they had weak anti-CHIKV activity (EC_50_ > 50 μM), unlike OBZ (Figure 3).

To get more insights into the stereoelectronic features important to OBZ’s activity, different physicochemical descriptors of OBZ and marketed analogs were calculated (Figure 3). OBZ showed the lowest values of dipole moment (DM), cLogP, molecular weight (MW), molecular surface area (MSA), and MV compared to its analogs. By contrast, electronic features, such as highest occupied molecular orbital (HOMO) and lowest unoccupied molecular orbital (LUMO) energies, were directly correlated to the antiviral activity, since OBZ showed the highest values. Besides, the increasing number of HBA and flexibility (RotB) seems to contribute to its anti-CHIKV activity.

The analysis of the molecular electrostatic potential (MEP) maps did not provide clear differences between the compounds (data not shown), but considerable changes were observed regarding the frontier orbitals HOMO and LUMO density maps (Figure 3). For OBZ and MBZ, HOMO density was concentrated over the benzimidazole ring, while the addition of substituents containing sulfur atoms (e.g., ABZ and FBZ) displaced the density of these orbitals. Regarding the LUMO map, the orbitalś density of OBZ was concentrated over the benzimidazole and carbamate moieties, as observed for ABZ and MBZ. However, the introduction of the benzoyl group in MBZ led to a drastic change in LUMO density, which was concentrated over the substituent group. Therefore, the concentration of HOMO and LUMO orbitals simultaneously over the benzimidazole moiety seems to be important for the antiviral activity of OBZ and its analogs.

### 2.4 Investigation of the Mechanism of Action of OBZ

**Figure 4:**
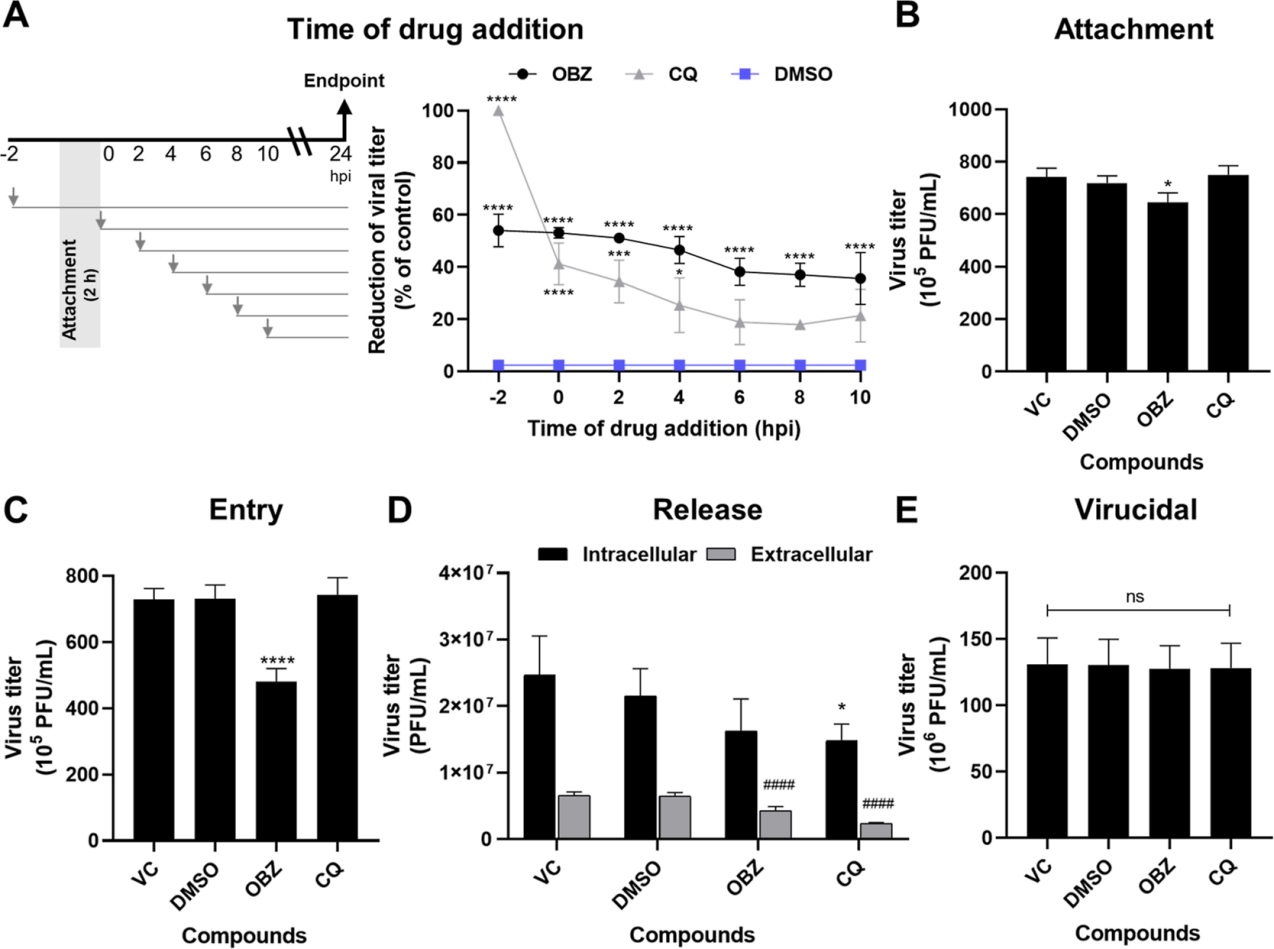
Mechanism of action studies of oxibendazole (OBZ) against CHIKV BRA/RJ/18 strain. The antiviral effects of OBZ (50 μM) were evaluated in a (A) time-of-addition assay, in which the compound was added at different times according to the left scheme. The inhibitory effects of OBZ were further evaluated at specific steps of virus replication, such as (B) attachment, (C) entry, and (D) release of new virus particles. (E) Virucidal effect of this compound was also evaluated after treating a suspension of 2 × 10^5^ plaque-forming units (PFU) for 2 h at 37 °C. The remaining infectivity of virus suspensions was evaluated. For all experiments, chloroquine (CQ, 100 μM) and DMSO (0.5%) as well as infected and untreated cells (virus control, VC) were used as controls. At the assays’ end points, cells were lysed by three cycles of freezing and thawing, and supernatants were tittered by plaque assays. Data are expressed as mean ± standard deviation (SD) (*n* = 3), and statistical analyses were carried out using the ANOVA method followed by the Tukey test. Statistical significance was analyzed with reference to the VC group, except for the time-of-addition assay in which the DMSO-treated group was used as reference. For panel D, asterisks and hash marks refer to the comparison of virus titers in intracellular and extracellular groups, respectively (*p < 0.05; ***p < 0.001; ****p < 0.0001; ####*p* < 0.0001; ns: not significant).

To get more insight into the mechanism of antiviral action of OBZ, we carried out a set of experiments. First, the time-of-addition assay was carried out to investigate at which step of virus replication this compound might act. The inhibition rates of this compound were maintained at approximately 55% when added until 4 hpi (Figure 4A). When added after this time, the antiviral effect of OBZ was reduced but kept around 40% until 10 hpi. Since no significant differences were observed after pre- and post-infection treatment, these results suggest that this compound did not protect cells from viral infection, unlike CQ.

Consequently, we evaluated the inhibitory activity of OBZ at specific steps of CHIKV replication, such as attachment and entry (Figure 4B,C). Although OBZ reduced virus titers significantly in the attachment assay, the inhibition rate was low (13.1%) compared to the infected and untreated group. Likewise, OBZ reduced virus titers significantly in the virus entry assay, but a higher inhibition rate was observed (33.8%). Also, the effects of this compound on CHIKV release were evaluated (Figure 4D). This compound was able to reduce virus titers in the extracellular medium significantly. However, the intracellular/extracellular ratio of OBZ (3.78) was similar to the ones observed for the DMSO (3.32) and VC (3.73) groups, suggesting that this compound does not interfere with virus egress. Finally, we investigated whether OBZ could directly inactivate CHIKV particles by treating virus suspensions before infection (Figure 4E). However, no virucidal effects were observed for this drug, indicating that it exerts its antiviral effects by blocking virus replication in host cells.

## 3 Discussion

Our group investigated key structural features for inhibiting CHIKV nsP2 protease by molecular modeling tools.^21^ Herein, we explored these features in the search for potential inhibitors within DrugBank libraries by conducting a hybrid virtual screening to identify new drug repurposing opportunities to fight CHIKV infections. Initially, based on our former findings, we developed a pharmacophore model that recovered all known inhibitors of the test set. The limited number of proven inhibitors and their low structural diversity limit the assessment of the pharmacophore models’ performance using other metrics, which could have compromised the selection of compounds with novel scaffolds as potential inhibitors. Yet, we included other validation steps throughout the virtual screening pipeline to improve our success rate. The employed pharmacophore model explored similar interactions as the ones reported in other works,^22,23^ but also the contacts with N1082 and H1083, which strongly suggest that our model comprised important characteristics to identify new antivirals.

Besides using the pharmacophore-based step, other tools, such as drug-like properties prediction, molecular docking and dynamics, and pharmaceutical analysis, were associated as a hybrid virtual screening campaign. Considering the limited accuracy of scoring functions in docking studies,^24^ we first assessed 10 different functions alone or in combination. The combination of smina, vinardo, and ad4_scoring functions yielded the highest AUC-ROC (0.72), demonstrating that this method could discriminate between active and inactive ligands and was further selected for target-based virtual screening.

A total of 5944 ligands were screened and the selected ones had drug-like properties and interacted with the catalytic residue C1013 as well as with other residues important for the binding of other inhibitors of nsP2 protease of CHIKV or other alphaviruses, such as N1011, A1046, Y1047, Y1079, N1082, and W1084.^21,25,26^ In the MD simulation step, the selected ligands showed smaller conformational changes than the inactive ligand and their binding to the nsP2 protease maintained similar movements as observed for complexes with known inhibitors. Since these are valuable properties to differentiate active and inactive ligands,^21^ these results indicate a favorable dynamic behavior for the enzyme’s inhibition. Finally, six virtual hits were selected and two of them were purchased for experimental validation: the anticancer drug, CPB, and the antiparasitic drug, OBZ.

We first evaluated their antiviral activity (50 μM) against the replication of the attenuated CHIKV 181-25 strain (Asian genotype) in Vero cells. CPB did not inhibit CHIKV replication, but OBZ reduced virus titers by 66.4%. Cytotoxicity assays confirmed that the antiviral activity observed was not due to cytotoxic-mediated effects, since this compound had a CC_50_ higher than 500 μM. Different CHIKV strains present different replication kinetics, lead to different pathogenesis, and result in different disease severities. Accordingly, similar treatments can show different antiviral efficacies against different CHIKV strains in vitro and in vivo.^27,28^ Consequently, we further investigated the antiviral effects of OBZ against a Brazilian isolate (BRA/RJ/18; ECSA genotype), which is a more virulent strain and is possibly more associated with chronic infections.^13^ Although the drug CQ showed a decreased activity against this virulent strain (48.5%), OBZ exhibited a strain-independent antiviral activity and maintained a similar inhibition rate (61.8%). This compound was nearly 4-fold more potent than CQ with EC_50_ values of 11.4 and 47.5 μM, respectively, and presented higher selectivity (SI > 44) than CQ (9.9).

OBZ is an approved antiparasitic drug that has been used to treat helminth infection in animals for over two decades.^29^ In preclinical studies with rats and mice, the 50% lethal dose (LD_50_) was not determined because no deaths were reported even after treatment with doses up to 10 g/kg.^30^ Clinical trials have demonstrated the safety of this drug due to the low frequency of side effects following a single oral dose of 400 mg,^31^ which reinforces the safety profile of this compound for further studies. To date, few drug repurposing opportunities have been described for treating CHIKV infections, such as sofosbuvir (EC_50_ = 2.7 μM; SI = 149),^32^ micafungin (EC_50_ = 20.63 μM; SI > 4.85),^33^ nitazoxanide (EC_50_ = 2.96 μM; SI = 8.45),^34^ suramin (EC_50_ = 21.50 μM; SI > 32.6),^35^ orlistat (EC_50_ = 0.82 μM; SI = 10.57),^36^ and telmisartan (EC_50_ = 15.34 μM; SI > 19.55).^37^ Among these drugs, sofosbuvir, suramin, and telmisartan were also demonstrated to ameliorate CHIKV infection in mice.^32,37,38^ Excitingly, OBZ had higher potency than micafungin, suramin, and telmisartan and was more selective than nitazoxanide and orlistat, but sofosbuvir had higher potency and selectivity. It is worth noting that CHIKV infection is a neglected disease that affects mainly middle- and low-income countries and the high costs associated with sofosbuvir may limit access to antiviral therapy,^39^ unlike OBZ that has lower costs and offers an interesting alternative.

Since OBZ is approved for animal use only, we also evaluated the antiviral activity of its marketed analogs, such as ABZ, MBZ, and FBZ. Despite structural similarities, none of them had good anti-CHIKV properties (EC_50_ > 50 μM). As benzimidazole derivatives have been described with anti-CHIKV activity in literature,^40,41^ we calculated some stereoelectronic properties of these compounds to better understand structural features important to their anti-CHIKV activity. We found some steric features (e.g., MW, MSA, and MV) inversely correlated to their antiviral activity, while flexibility (RotB), HBA, *E*_HOMO,_ and *E*_LUMO_ contributed favorably to their activity. Also, the simultaneous concentration of HOMO and LUMO orbitals over the benzimidazole moiety seems to play a key role in their antiviral activity. However, in this work, only four compounds were evaluated, which is a limited number of compounds to establish a reliable structure–activity relationship. Yet, the structural trend observed here might open a new venue for the design of novel OBZ analogs with antiviral activity. For instance, the introduction of HBA groups with lower volumes as substituents attached to the benzimidazole moiety likely favors the anti-CHIKV profile of new derivatives and should be explored in the future as an alternative to drug repurposing.

Considering the antiviral potential of OBZ, we further investigated its mechanism of action. OBZ showed weak-to-moderate inhibitory effects on virus attachment (13.1%) and entry (33.8%). Lysosomotropic drugs, like CQ, exhibit reversible antiviral effects if removed within 90 min after alphavirus infection,^42^ as observed in our assays. Thus, our results suggest that OBZ interferes with virus entry through mechanisms other than altering endosomal pH.

In addition, no effects were observed on virus particle release, cell protection, or viral particles (virucidal effect). These results agree with the time-of-addition assay, in which OBZ showed the highest effects (∼55%) when added until 4 hpi, which in turn suggests that this compound acts at post-entry steps, such as virus replication as well. To the best of our knowledge, known CHIKV nsP2 protease inhibitors show similar time-course effects on CHIKV replication reaching the highest antiviral activity when added at the same time,^23,37,43^ which suggests that OBZ could be an inhibitor of this protein as predicted. However, other experiments, such as enzymatic or resistance assays, must be conducted to confirm this hypothesis. Additionally, telmisartan is a proven CHIKV nsP2 inhibitor^44^ that exhibits multiple mechanisms of action by targeting different steps of virus replication as well as host factors.^37^ In fact, OBZ is known to bind β-tubulin and block microtubule formation in helminths,^45^ which was demonstrated to be important for CHIKV replication.^46^ Therefore, one could not exclude that this mechanism is not involved in the antiviral activity of OBZ and a more in-depth characterization of its mechanism of action remains to be performed.

Collectively, our findings point to the antiviral potential of the antiparasitic drug OBZ, which deserves further in vivo validation as a drug candidate against CHIKV. However, poor solubility is a limitation of the use of benzimidazole antihelmintics. Yet, different pharmaceutical strategies and technologies are available and under discussion to overcome this issue and enhance the oral bioavailability of these drugs in the future.^47^ Moreover, the antiviral activity of OBZ has already been reported against human herpesvirus 8 (EC_50_ = 1.5 μM)^48^ and Zika virus (EC_50_ = 0.7 μM),^49^ pointing to its broad-antiviral spectrum that is highly desirable for treating remerging and emerging viruses.^50^

## 4 Conclusions

In this work, we conducted a hybrid virtual screening campaign to search for new drug repurposing opportunities to treat CHIKV infections. This strategy has proven to be valuable as we screened thousands of compounds within DrugBank libraries and identified six drugs as potential CHIKV nsP2 protease inhibitors. Two virtual hits, CPB and OBZ, were selected for in vitro validation. Interestingly, the antiparasitic drug OBZ showed low cytotoxicity and high antiviral activity against two CHIKV strains as well as high selectivity. Despite the promising profile of OBZ, the three marketed drugs that are analogs of OBZ, namely, ABZ, MBZ, and FBZ, exhibited weak antiviral activity. The analysis of their stereoelectronic features suggested some properties to be related to the OBZ’s antiviral profile, which could guide the synthesis of new analogs and aid the design of novel derivatives with improved activity. Finally, the investigation of the mechanism of action of OBZ indicated that this drug acts at early and post-entry events of virus replication. Taken together, this is the first report of the anti-CHIKV activity of the drug OBZ and our results encourage further in vivo assays to validate it as a drug candidate to fight Chikungunya fever.

## 5 Materials and Methods

### 5.1 Computational Studies

#### 5.1.1 Library Preparation

In this study, we selected different DrugBank libraries available in the ZINC15 database, such as approved human and veterinary drugs (2582 and 261 compounds, respectively) nutraceuticals (122 compounds), withdrawn (331 compounds), and investigational drugs (2648 compounds). The 3D structure of the compounds was retrieved from the ZINC15 database and then was protonated at pH 7.4 using the OpenBabel 2.4.1 program. The structures were then submitted to 2500 cycles of energy minimization using the Steepest descent method, followed by 250 cycles of energy minimization using the conjugated gradient method employing the MMFF94 force field in the same program.

#### 5.1.2 Pharmacophore-based Virtual Screening

Pharmacophore modeling was performed according to previous findings reported by our group regarding the binding mode and behavior of known inhibitors with the CHIKV nsP2 protease.^21^ The same set of active ligands (15 compounds) was used in this study for the development of pharmacophore models. Two approaches were conducted to construct pharmacophore models: (I) the models were manually constructed using the Pharmit server based on the binding mode of the most active inhibitors (*RRS*)-**C5** and **C12** with the nsP2 protease, and (II) the models were automatically constructed using the Pharmagist server^51^ based on the superimposition of the binding mode of different inhibitors, such as (a) more active ligands [i.e., (*SS*)-**C7**, (*S*)-**C9**, and **C12**], (b) structurally similar ligands with a defined stereochemistry [i.e., (*RRS*)-**C5**, (*SS*)-**C7**, and (*S*)-**C9**], and (c) more active ligands with a defined stereochemistry [i.e., (*RRS*)-**C5**, (*S*)-**C9**, and **C12**]. The radius of pharmacophore groups was kept as default unless otherwise stated (e.g., aromatic and hydrophobic groups = 1.0 Å and hydrogen-bond acceptor and donor groups = 0.5 Å).

The performance of each model was evaluated according to its recovery rate, which refers to the ability of the pharmacophore models to select the known ligands within a set of active ligands. All inhibitors reported in our previous work were included,^21^ except the ones used in the construction of the evaluated model. The selected pharmacophore model (model F) was used to screen the DrugBank libraries (5944 compounds) using the Pharmit server.^52^ To avoid redundancy, only one conformation of each compound was selected.

#### 5.1.3 Drug-Like Properties Filtering

All compounds obtained in the pharmacophore-based virtual screening were submitted to the FAF-Drugs4 server^53^ to remove duplicates. Also, the obtained withdrawn and investigational drugs were further selected using the drug-like and Pan Assays Interferences Compounds (PAINS) filters in the same server.

#### 5.1.4 Target-Based Virtual Screening

To validate and select the scoring function for target-based virtual screening, different scoring functions were evaluated for their ability to discriminate active and inactive ligands of the CHIKV nsP2 protease. The active ligands described in our previous work^21^ were included. For each active ligand, 50 decoys were selected on the ZINC database using the DUD-E server, totaling 15 active ligands and 750 decoys. The 3D structure of the decoys was constructed using the RDKit node available in the Knime 3.4 program. Further prediction of protonation states and structural optimization steps were carried out as described for the preparation of the DrugBank libraries, with a few modifications. After the geometry optimization by the Steepest descent method, the structures were submitted to a conformational analysis, in which 200 conformations were evaluated using the weighted method. The lowest-energy conformer was further optimized by 2500 cycles of energy minimization using the conjugated gradient method. Finally, partial charges were calculated using the semiempirical EEM method based on DFT-B3LYP/6-311G/NPA using the OpenBabel program.

All compounds were docked within the active site of the CHIKV nsP2 protease (PDB code 3TRK) using the Autodock 4.2.6 and Autodock Tools 1.5.7 programs, as validated previously.^21^ The conformation with the lowest binding energy of each ligand was retrieved and rescored with different scoring functions, such as Autodock Vina (available with Autodock Vina 1.1.2 program), smina, vinardo, ad4_scoring (available with smina program), Chemscore, ASP, ChemPLP, Goldscore (available with Gold5.4 program), and DSX. The area under the receiver operating characteristic (AUC-ROC) was calculated using the Screening Explorer server.^54^ The consensus results of scoring functions with AUC-ROC ≥ 0.6 were carried out using the average *Z*-score method calculated as described by Empereur-Mot and co-workers.^54^

For the target-based virtual screening steps, the selected compounds were docked within the active site of CHIKV nsP2 protease using the Autodock 4.2.6 program. Then, the lowest binding energy conformation was rescored using the average *Z*-score obtained from ad4_scoring, smina, and vinardo scoring functions. The compounds were ranked in descending order according to their *Z*-score values and 20% of top-scoring ligands had their binding mode visually inspected. As selection criteria, we considered the interaction with the catalytic dyad (C1013 and H1083) as well as the interactions with the subsites of the active site of the enzyme, as observed for the known inhibitors.

#### 5.1.5 Pharmaceutical Filtering

The selected drugs were further analyzed based on pharmaceutical criteria, such as side effects or administration routes described in the literature to obtain feasible compounds for the treatment of CHIKV infections. For instance, drugs with serious neurological side effects or with intravenous administration only were removed.

#### 5.1.6 Molecular Dynamics Simulation

The CHIKV nsP2 protease in complex with the selected drugs obtained from molecular docking studies was submitted MD simulation for 30 ns at 310 K using the AMBER16 and AmberTools17 programs, as described elsewhere.^21^ The RMSD of the ligands with reference to their initial conformation and their effects on the protein’s movements by calculating the DCCM were calculated using the cpptraj module.

#### 5.1.7 Prediction of Stereoelectronic Properties of OBZ and Its Analogs

The 3D structure of OBZ and its analogs albendazole (ABZ), mebendazole (MBZ), and fenbendazole (FBZ) were constructed and optimized using the Spartan’10 program (Wave function Inc. Irvine, CA). The structure was initially submitted to a conformational analysis in vacuum using molecular mechanics and the MMFF force field. The lowest-energy conformer was geometrically optimized using the semiempirical RM1 method, followed by ab initio calculation using the DFT method with the B3LYP/6-31* basis set. Finally, different stereoelectronic properties were calculated, such as molecular weight (MW), *n*-octanol/water partition coefficient (cLogP), molecular surface area (MSA) and volume (MV), polar surface area (PSA), dipole moment (DM), the HOMO and LUMO energies (*E*_HOMO_ and *E*_LUMO_, respectively). MEP maps as well as HOMO and LUMO density maps were also calculated. In addition, the number of hydrogen bond acceptor and donor groups (HBA and HBD, respectively) and the number of rotatable bonds (RotB) were calculated using the Molinspiration cheminformatics server (Slovensky Grob, Slovak Republic, 2021, http://www.molinspiration.com).

### 5.2 Experimental Studies

#### 5.2.1 Cells, Viruses, and Compounds

Vero cells (ATCC CCL-81) were grown in Dulbecco’s modified Eagle medium supplemented with 5% fetal bovine serum (FBS), 100 μg/mL streptomycin, 100 U/mL penicillin (Gibco), and 2.5 μg/mL amphotericin B (Sigma-Aldrich, Brazil). Cells were kept under these conditions in all experiments unless otherwise stated. The CHIKV 181–25 strain and the clinical isolate CHIKV BRA/RJ/18 (GenBank accession MK910739) were used in this work. The CHIKV 181-25 is a live-attenuated strain derived from a human isolate belonging to the Asian genotype,^55^ while the BRA/RJ/18 isolate was isolated from a Brazilian patient and belongs to the ECSA genotype as described previously by our group.^56^ CHIKV strains were handled following BSL 2 and BSL 3 protocols, respectively.

Capecitabine (CPB), oxibendazole (OBZ), and its analogs albendazole (ABZ), mebendazole (MBZ), and fenbendazole (FBZ) were obtained from AK Scientific (>97% purity), while chloroquine (CQ; >98% purity) was purchased from Sigma-Aldrich (Brazil). Compounds were stored at −20 °C in dimethyl sulfoxide (DMSO) stock solutions, except for CQ which was dissolved in sterile water.

#### 5.2.2 Antiviral Screening of the Virtual Hits CPB and OBZ Against the CHIKV 181–25 Replication

Vero cells (2 × 10^5^ cells/well) seeded on 24-well plates were infected with the CHIKV 181–25 strain at an MOI of 1.0 for 2 h at 37 °C and 5% CO_2_ to allow for virus attachment. After this, the virus inoculum was removed, cells were washed with PBS (pH 7.4) and subsequently treated with 50 μM of CPB or OBZ for 24 h at 37 °C and 5% CO_2_. DMSO (0.5%) was used as vehicle control, while CQ (50 μM) was used as a positive control due to its in vitro antiviral activity against CHIKV.^57^ At 24 h post-infection (hpi), cells were lysed by three cycles of freezing and thawing, and supernatants were harvested for virus titer determination by plaque assay.

For plaque assays, confluent monolayers of Vero cells were infected with serially 10-fold dilutions for 2 h at 37 °C and 5% CO_2_. Then, the virus inoculum was discarded, and cells were overlaid with fresh medium supplemented with 5% FBS and 1.5% methylcellulose and incubated for further 72 h at 37 °C and 5% CO_2_. Finally, the overlay medium was removed, and cells were fixed and stained with 10% formaldehyde and 0.2% crystal violet. Plaques were counted, and virus titers were determined as PFU/mL. Inhibition rates were determined by comparing the number of PFU of infected and treated cells and infected and untreated cells (100% infectivity).

#### 5.2.3 Cytotoxicity Evaluation

The effects of OBZ and its analogs ABZ, MBZ, and FBZ on Vero cell viability were measured by MTT assay. Vero cells (2 × 10^4^ cells/well) were treated with different concentrations (10, 50, 125, 250, and 500 μM) of the compounds for 72 h at 37 °C and 5% CO_2_ atmosphere. CQ (62.5, 125, 250, 500, and 1000 μM) were also evaluated. Then, the MTT assay was performed as described previously.^58^ The concentration of the compounds needed to decrease cell viability by half (CC_50_) was calculated from dose–response curves using the GraphPad Prism 8 software.

#### 5.2.4 Antiviral Evaluation of OBZ and Its Analogs Against a Brazilian CHIKV Isolate

The antiviral activity of OBZ and its analogs (ABZ, MBZ, and FBZ) was evaluated against the replication of CHIKV BRA/RJ/18 in Vero cells as described for the CHIKV 181–25 strain. To determine their potency, infected cells (MOI 1) were treated with different concentrations of OBZ (1.0, 3.125, 12.5, 25, 50, and 100 μM) for 24 h at 37 °C and 5% CO_2_ atmosphere. ABZ, MBZ, and FBZ were assessed at the same conditions and concentrations, except for 100 μM. CQ was included as a positive control. At 24 h, cells were lysed, and supernatants were harvested. Virus titers were determined by plaque assays as mentioned above, except that infected cells were fixed and stained at 48 hpi. Inhibition rates were calculated with reference to the virus control group (VC). The concentration required to inhibit 50% of virus replication (EC_50_) was estimated by nonlinear regression. The selectivity index (SI) was calculated as the ratio between CC_50_ and EC_50_ values.

#### 5.2.5 Time-of-Drug Addition Assay

Vero cells (2 × 10^5^ cells/well) were infected with CHIKV BRA/RJ/18 (MOI 1) for 2 h at 37 °C and 5% CO_2_. After attachment, the virus inoculum was removed, and cells were treated with OBZ (50 μM) at different times post-infection (0, 2, 4, 6, 8, and 10 hpi). A 2 h pretreatment was also carried out (−2 hpi). CQ (100 μM), and DMSO (0.5%) were used as controls. Once added, the compounds were kept in the medium until the end point. At 24 hpi, cells were lysed by three cycles of freezing and thawing, and supernatants were harvested and tittered by plaque assays. Inhibition rates were calculated related to the infected and untreated group.

#### 5.2.6 Attachment Assay

Vero cells (2 × 10^5^ cells/well) were prechilled at 4 °C for 1 h and, then, infected with CHIKV BRA/RJ/18 (MOI 1) for 1 h at the same temperature. OBZ (50 μM) was added during this time. As controls, CQ (100 μM) and DMSO (0.5%) were used. After attachment, virus inoculum and compounds were removed, cells were thoroughly washed with cold PBS (pH 7.4) and fresh medium was added. Cells were incubated for 24 h at 37 °C under a 5% CO_2_ atmosphere. At 24 hpi, cells were lysed by three cycles of freezing and thawing, and virus titers of collected supernatants were determined by plaque assays.

#### 5.2.7 Entry Assay

Prechilled Vero cells (2 × 10^5^ cells/well) were infected with CHIKV BRA/RJ/18 at an MOI of 1.0 for 1 h at 4 °C. Then, the virus inoculum was removed, cells were washed twice with cold PBS (pH 7.4), fresh medium was added in the presence or absence of OBZ (50 μM), and cells were incubated for 1 h at 37 °C and 5% CO_2_ to allow for virus entry. CQ (100 μM) and DMSO (0.5%) were also evaluated. After this, the medium was removed, and cells were washed with acidic PBS (pH 3.0) for 1 min, followed by three washes with PBS (pH 7.4). Finally, fresh medium was added, and cells were further incubated for 23 h at 37 °C under a 5% CO_2_ atmosphere. At 24 hpi, cells were lysed by three cycles of freezing and thawing, supernatants were collected, and virus titers were quantified by plaque assays.

#### 5.2.8 Virus Release Assay

Vero cells (2 × 10^5^ cells/well) were infected with CHIKV BRA/RJ/18 strain (MOI = 1) for 2 h at 37 °C and 5% CO_2_. After attachment, the virus inoculum was removed, cells were thoroughly washed with PBS (pH 7.4) and fresh medium was added in the presence or absence of OBZ (50 μM). CQ (100 μM) and DMSO (0.5%) were included as controls. Posteriorly, cells were incubated for 6 h under the same conditions. At 6 hpi, supernatants were collected as the extracellular content. Then, fresh medium was added to cells which were subsequently lysed by three cycles of freezing and thawing, and the intracellular content was collected. Virus titers in both extracellular and intracellular contents were quantified by plaque assays.

#### 5.2.9 Virucidal Assay

The virucidal assay was carried out using the CHIKV BRA/RJ/18 strain. Virus suspensions containing 2 × 10^5^ PFU were treated with OBZ (50 μM), CQ (100 μM), or DMSO (0.5%) for 2 h at 37 °C. To avoid any interference in virus replication in cells, virus suspensions were diluted (1:100) and used to infect Vero cells (2 × 10^5^ cells/well) for 2 h at 37 °C and 5% CO_2_. After attachment, virus inoculum was removed, fresh medium was added, and cells were incubated for 24 h under the same conditions. At 24 hpi, cells were lysed by three cycles of freezing and thawing, and the supernatants were harvested for plaque assays.

#### 5.2.10 Statistical Analyses

In vitro data are shown as mean ± SD of three independent experiments run in triplicate. Statistical analyses were conducted using the ANOVA method followed by the Tukey test for multiple comparisons within the GraphPad Prism v. 8.0 program. Differences were considered statistically significant when *p* ≤ 0.05.

## Data Availability

The data underlying this study are available in the published article and its Supporting Information.

## Supporting Information Available

Pharmacophore modeling and virtual screening validation results, including cytotoxic and antiviral dose–response curves of oxibendazole (PDF)

## References

[ref1] Singh A.; Kumar A.; Yadav R.; Uversky V. N.; Giri R. Deciphering the Dark Proteome of Chikungunya Virus. Sci. Rep. 2018, 8 (1), 5822 10.1038/s41598-018-23969-0. 29643398 PMC5895634

[ref2] Cavalcanti T. Y. V. d. L.; Pereira M. R.; Paula S. O. d.; Franca R. F. d. O. A Review on Chikungunya Virus Epidemiology, Pathogenesis and Current Vaccine Development. Viruses 2022, 14 (5), 969 10.3390/V14050969. 35632709 PMC9147731

[ref3] Rougeron V.; Sam I. C.; Caron M.; Nkoghe D.; Leroy E.; Roques P. Chikungunya, a Paradigm of Neglected Tropical Disease That Emerged to Be a New Health Global Risk. J. Clin. Virol. 2015, 64, 144–152. 10.1016/j.jcv.2014.08.032. 25453326

[ref4] Casulli A. New Global Targets for NTDs in the WHO Roadmap 2021–2030. PLoS Negl. Trop. Dis. 2021, 15 (5), e0009373 10.1371/journal.pntd.0009373. 33983940 PMC8118239

[ref5] Grabenstein J. D.; Tomar A. S. Global Geotemporal Distribution of Chikungunya Disease, 2011–2022. Travel Med. Infect. Dis. 2023, 54, 102603 10.1016/j.tmaid.2023.102603. 37307983

[ref6] de Souza W. M.; Ribeiro G. S.; de Lima S. T. S.; de Jesus R.; Moreira F. R. R.; Whittaker C.; Sallum M. A. M.; Carrington C. V. F.; Sabino E. C.; Kitron U.; Faria N. R.; Weaver S. C. Chikungunya: A Decade of Burden in the Americas. Lancet Reg. Heal. Am. 2024, 30, 100673 10.1016/J.LANA.2023.100673. PMC10820659 38283942

[ref7] Souza W. M. d.; de Lima S. T. S.; Simões Mello L. M.; Candido D. S.; Buss L.; Whittaker C.; Claro I. M.; Chandradeva N.; Granja F.; de Jesus R.; Lemos P. S.; Toledo-Teixeira D. A.; Barbosa P. P.; Firmino A. C. L.; Amorim M. R.; Duarte L. M. F.; Pessoa I. B.; Forato J.; Vasconcelos I. L.; Maximo A. C. B. M.; Araújo E. L. L.; Perdigão Mello L.; Sabino E. C.; Proença-Módena J. L.; Faria N. R.; Weaver S. C. Spatiotemporal Dynamics and Recurrence of Chikungunya Virus in Brazil: An Epidemiological Study. Lancet 2023, 4 (5), e319–e329. 10.1016/S2666-5247(23)00033-2. PMC10281060 37031687

[ref8] ECDC. Chikungunya worldwide overview https://www.ecdc.europa.eu/en/chikungunya-monthly (accessed Feb 7, 2024).

[ref9] Harris E. WHO: Concerning Spread of Dengue, Chikungunya in Latin America. JAMA 2023, 329 (16), 1341 10.1001/jama.2023.5624. 37017997

[ref10] George A. M.; Ansumana R.; de Souza D. K.; Niyas V. K. M.; Zumla A.; Bockarie M. J. Climate Change and the Rising Incidence of Vector-Borne Diseases Globally. Int. J. Infect. Dis. 2024, 139, 143–145. 10.1016/j.ijid.2023.12.004. 38096974

[ref11] Kraemer M. U. G.; Reiner R. C.; Brady O. J.; Messina J. P.; Gilbert M.; Pigott D. M.; Yi D.; Johnson K.; Earl L.; Marczak L. B.; Shirude S.; Davis Weaver N.; Bisanzio D.; Perkins T. A.; Lai S.; Lu X.; Jones P.; Coelho G. E.; Carvalho R. G.; Van Bortel W.; Marsboom C.; Hendrickx G.; Schaffner F.; Moore C. G.; Nax H. H.; Bengtsson L.; Wetter E.; Tatem A. J.; Brownstein J. S.; Smith D. L.; Lambrechts L.; Cauchemez S.; Linard C.; Faria N. R.; Pybus O. G.; Scott T. W.; Liu Q.; Yu H.; Wint G. R. W.; Hay S. I.; Golding N. Past and Future Spread of the Arbovirus Vectors Aedes Aegypti and Aedes Albopictus. Nat. Microbiol. 2019, 4 (5), 854–863. 10.1038/s41564-019-0376-y. 30833735 PMC6522366

[ref12] Vairo F.; Haider N.; Kock R.; Ntoumi F.; Ippolito G.; Zumla A. Chikungunya: Epidemiology, Pathogenesis, Clinical Features, Management, and Prevention. Infect. Dis. Clin. North Am. 2019, 33 (4), 1003–1025. 10.1016/j.idc.2019.08.006. 31668189

[ref13] Paixão E. S.; Rodrigues L. C.; Costa M. d. C. N.; Itaparica M.; Barreto F.; Gérardin P.; Teixeira M. G. Chikungunya Chronic Disease: A Systematic Review and Meta-Analysis. Trans. R. Soc. Trop. Med. Hyg. 2018, 112 (7), 301–316. 10.1093/trstmh/try063. 30007303

[ref14] Mehta R.; Gerardin P.; de Brito C. A. A.; Soares C. N.; Ferreira M. L. B.; Solomon T. The Neurological Complications of Chikungunya Virus: A Systematic Review. Rev. Med. Virol. 2018, 28 (3), 1–24. 10.1002/rmv.1978. PMC5969245 29671914

[ref15] Costa L. B.; Barreto F. K. d. A.; Barreto M. C. A.; Santos T. H. P. d.; Andrade M. d. M. O. d.; Farias L. A. B. G.; Freitas A. R. R. d.; Martinez M. J.; Cavalcanti L. P. d. G. Epidemiology and Economic Burden of Chikungunya: A Systematic Literature Review. Trop. Med. Infect. Dis. 2023, 8 (6), 301 10.3390/TROPICALMED8060301. 37368719 PMC10302198

[ref16] Mullard A. FDA Approves First Chikungunya Vaccine. Nat. Rev. Drug Discovery 2023, 23 (1), 8 10.1038/D41573-023-00201-X. 38049467

[ref17] Manzoor K. N.; Javed F.; Ejaz M.; Ali M.; Mujaddadi N.; Khan A. A.; Khattak A. A.; Zaib A.; Ahmad I.; Saeed W. K.; Manzoor S. The Global Emergence of Chikungunya Infection: An Integrated View. Rev. Med. Virol. 2022, 32 (3), e2287 10.1002/rmv.2287. 34428335

[ref18] Pushpakom S.; Iorio F.; Eyers P. A.; Escott K. J.; Hopper S.; Wells A.; Doig A.; Guilliams T.; Latimer J.; McNamee C.; Norris A.; Sanseau P.; Cavalla D.; Pirmohamed M. Drug Repurposing: Progress, Challenges and Recommendations. Nat. Rev. Drug Discovery 2019, 18 (1), 41–58. 10.1038/nrd.2018.168. 30310233

[ref19] Saisawang C.; Sillapee P.; Sinsirimongkol K.; Ubol S.; Smith D. R.; Ketterman A. J. Full Length and Protease Domain Activity of Chikungunya Virus NsP2 Differ from Other Alphavirus NsP2 Proteases in Recognition of Small Peptide Substrates. Biosci. Rep. 2015, 35 (3), e00196 10.1042/BSR20150086. 26182358 PMC4445351

[ref20] Fros J. J.; van der Maten E.; Vlak J. M.; Pijlman G. P. The C-Terminal Domain of Chikungunya Virus NsP2 Independently Governs Viral RNA Replication, Cytopathicity, and Inhibition of Interferon Signaling. J. Virol. 2013, 87 (18), 10394–10400. 10.1128/JVI.00884-13. 23864632 PMC3753987

[ref21] Rabelo V. W.-H.; Paixão I. C. N. d. P.; Abreu P. A. Structural Insights into the Inhibition of the NsP2 Protease from Chikungunya Virus by Molecular Modeling Approaches. J. Mol. Model. 2022, 28 (10), 311 10.1007/S00894-022-05316-3. 36097090

[ref22] Bassetto M.; De Burghgraeve T.; Delang L.; Massarotti A.; Coluccia A.; Zonta N.; Gatti V.; Colombano G.; Sorba G.; Silvestri R.; Tron G. C.; Neyts J.; Leyssen P.; Brancale A. Computer-Aided Identification, Design and Synthesis of a Novel Series of Compounds with Selective Antiviral Activity against Chikungunya Virus. Antiviral Res. 2013, 98 (1), 12–18. 10.1016/j.antiviral.2013.01.002. 23380636

[ref23] Das P. K.; Puusepp L.; Varghese F. S.; Utt A.; Ahola T.; Kananovich D. G.; Lopp M.; Merits A.; Karelson M. Design and Validation of Novel Chikungunya Virus Protease Inhibitors. Antimicrob. Agents Chemother. 2016, 60 (12), 7382–7395. 10.1128/AAC.01421-16. 27736770 PMC5119020

[ref24] Guedes I. A.; Pereira F. S. S.; Dardenne L. E. Empirical Scoring Functions for Structure-Based Virtual Screening: Applications, Critical Aspects, and Challenges. Front. Pharmacol 2018, 9, 1089 10.3389/fphar.2018.01089. 30319422 PMC6165880

[ref25] Rabelo V. W.-H.; Paixão I. C. N. d. P.; Abreu P. A. Targeting Chikungunya Virus by Computational Approaches: From Viral Biology to the Development of Therapeutic Strategies. Expert Opin. Ther. Targets 2020, 24 (1), 63–78. 10.1080/14728222.2020.1712362. 31914351

[ref26] Hu X.; Compton J. R.; Leary D. H.; Olson M. A.; Lee M. S.; Cheung J.; Ye W.; Ferrer M.; Southall N.; Jadhav A.; Morazzani E. M.; Glass P. J.; Marugan J.; Legler P. M. Kinetic, Mutational, and Structural Studies of the Venezuelan Equine Encephalitis Virus Nonstructural Protein 2 Cysteine Protease. Biochemistry 2016, 55 (21), 3007–3019. 10.1021/acs.biochem.5b00992. 27030368 PMC5290728

[ref27] Julander J. G.; Dagley A.; Gebre M.; Komeno T.; Nakajima N.; Smee D. F.; Furuta Y. Strain-Dependent Disease and Response to Favipiravir Treatment in Mice Infected with Chikungunya Virus. Antiviral Res. 2020, 182, 104904 10.1016/j.antiviral.2020.104904. 32791074 PMC7543030

[ref28] Hucke F. I. L.; Bestehorn-Willmann M.; Bassetto M.; Brancale A.; Zanetta P.; Bugert J. J. CHIKV Strains Brazil (Wt) and Ross (Lab-Adapted) Differ with Regard to Cell Host Range and Antiviral Sensitivity and Show CPE in Human Glioblastoma Cell Lines U138 and U251. Virus Genes 2022, 58 (3), 188–202. 10.1007/s11262-022-01892-x. 35347588 PMC8960095

[ref29] Horton J. Human Gastrointestinal Helminth Infections: Are They Now Neglected Diseases?. In Trends in Parasitology; Elsevier Ltd., 2003; pp 527–531. 10.1016/j.pt.2003.09.007. 14580965

[ref30] Overgaauw P. A. M.; Boersema J. H. Anthelmintic Efficacy of Oxibendazole against Some Important Nematodes in Dogs and Cats. Vet. Q. 1998, 20 (2), 69–72. 10.1080/01652176.1998.9694842. 9563164

[ref31] GlaxoSmithKline. An open, non comparative study to assess the safety and efficacy, safety and tolerability of a single 400mg PO dose of oxibendazole in intestinal helminth infections in adults. GSK Study ID:30310/004 https://www.gsk-studyregister.com/en/trial-details/?id=30310/004 (accessed Sep 15, 2020).

[ref32] Ferreira A. C.; Reis P. A.; de Freitas C. S.; Sacramento C. Q.; Villas Bôas Hoelz L.; Bastos M. M.; Mattos M.; Rocha N.; Gomes de Azevedo Quintanilha I.; da Silva Gouveia Pedrosa C.; Rocha Quintino Souza L.; Correia Loiola E.; Trindade P.; Rangel Vieira Y.; Barbosa-Lima G.; de Castro Faria Neto H. C.; Boechat N.; Rehen S. K.; Brüning K.; Bozza F. A.; Bozza P. T.; Souza T. M. L. Beyond Members of the Flaviviridae Family, Sofosbuvir Also Inhibits Chikungunya Virus Replication. Antimicrob. Agents Chemother. 2019, 63, ( (2), ), e01389-18. 10.1128/AAC.01389-18. 30455237 PMC6355571

[ref33] Ho Y. J.; Liu F. C.; Yeh C. T.; Yang C. M.; Lin C. C.; Lin T. Y.; Hsieh P. S.; Hu M. K.; Gong Z.; Lu J. W. Micafungin Is a Novel Anti-Viral Agent of Chikungunya Virus through Multiple Mechanisms. Antiviral Res. 2018, 159, 134–142. 10.1016/j.antiviral.2018.10.005. 30300716

[ref34] Wang Y. M.; Lu J. W.; Lin C. C.; Chin Y. F.; Wu T. Y.; Lin L. I.; Lai Z. Z.; Kuo S. C.; Ho Y. J. Antiviral Activities of Niclosamide and Nitazoxanide against Chikungunya Virus Entry and Transmission. Antiviral Res. 2016, 135, 81–90. 10.1016/j.antiviral.2016.10.003. 27742486 PMC7126800

[ref35] Ho Y. J.; Wang Y. M.; Lu J. W.; Wu T. Y.; Lin L. I.; Kuo S. C.; Lin C. C. Suramin Inhibits Chikungunya Virus Entry and Transmission. PLoS One 2015, 10 (7), e0133511 10.1371/journal.pone.0133511. 26208101 PMC4514758

[ref36] Karlas A.; Berre S.; Couderc T.; Varjak M.; Braun P.; Meyer M.; Gangneux N.; Karo-Astover L.; Weege F.; Raftery M.; Schönrich G.; Klemm U.; Wurzlbauer A.; Bracher F.; Merits A.; Meyer T. F.; Lecuit M. A Human Genome-Wide Loss-of-Function Screen Identifies Effective Chikungunya Antiviral Drugs. Nat. Commun. 2016, 7, 11320 10.1038/ncomms11320. 27177310 PMC4865845

[ref37] De S.; Mamidi P.; Ghosh S.; Keshry S. S.; Mahish C.; Pani S. S.; Laha E.; Ray A.; Datey A.; Chatterjee S.; Singh S.; Mukherjee T.; Khamaru S.; Chattopadhyay S.; Subudhi B. B.; Chattopadhyay S. Telmisartan Restricts Chikungunya Virus Infection In Vitro and In Vivo through the AT1/PPAR-γ/MAPKs Pathways. Antimicrob. Agents Chemother. 2022, 66 (1), e01489–e01521. 10.1128/AAC.01489-21. 34748384 PMC8765259

[ref38] Kuo S. C.; Wang Y. M.; Ho Y. J.; Chang T. Y.; Lai Z. Z.; Tsui P. Y.; Wu T. Y.; Lin C. C. Suramin Treatment Reduces Chikungunya Pathogenesis in Mice. Antiviral Res. 2016, 134, 89–96. 10.1016/j.antiviral.2016.07.025. 27577529

[ref39] Barber M. J.; Gotham D.; Khwairakpam G.; Hill A. Price of a Hepatitis C Cure: Cost of Production and Current Prices for Direct-Acting Antivirals in 50 Countries. J. Virus Erad. 2020, 6 (3), 100001 10.1016/j.jve.2020.06.001. 33251019 PMC7646676

[ref40] Mishra P.; Kumar A.; Mamidi P.; Kumar S.; Basantray I.; Saswat T.; Das I.; Nayak T. K.; Chattopadhyay S. S.; Subudhi B. B.; Chattopadhyay S. S. Inhibition of Chikungunya Virus Replication by 1-[(2-Methylbenzimidazol-1-Yl) Methyl]-2-Oxo-Indolin-3-Ylidene] Amino] Thiourea(MBZM-N-IBT). Sci. Rep. 2016, 6, 20122 10.1038/srep20122. 26843462 PMC4740769

[ref41] Wada Y.; Orba Y.; Sasaki M.; Kobayashi S.; Carr M. J.; Nobori H.; Sato A.; Hall W. W.; Sawa H. Discovery of a Novel Antiviral Agent Targeting the Nonstructural Protein 4 (NsP4) of Chikungunya Virus. Virology 2017, 505, 102–112. 10.1016/j.virol.2017.02.014. 28236746

[ref42] Cassell S.; Edwards J.; Brown D. T. Effects of Lysosomotropic Weak Bases on Infection of BHK-21 Cells by Sindbis Virus. J. Virol. 1984, 52 (3), 857–864. 10.1128/jvi.52.3.857-864.1984. 6492263 PMC254606

[ref43] Singh H.; Mudgal R.; Narwal M.; Kaur R.; Singh V. A.; Malik A.; Chaudhary M.; Tomar S. Chikungunya Virus Inhibition by Peptidomimetic Inhibitors Targeting Virus-Specific Cysteine Protease. Biochimie 2018, 149, 51–61. 10.1016/j.biochi.2018.04.004. 29635044

[ref44] Tripathi P. K.; Soni A.; Singh Yadav S. P.; Kumar A.; Gaurav N.; Raghavendhar S.; Sharma P.; Sunil S.; Ashish; Jayaram B.; Patel A. K. Evaluation of Novobiocin and Telmisartan for Anti-CHIKV Activity. Virology 2020, 548, 250–260. 10.1016/j.virol.2020.05.010. 32791353

[ref45] Abongwa M.; Martin R. J.; Robertson A. P. A Brief Review on the Mode of Action of Antinematodal Drugs. Acta Veterinaria 2017, 67, 137–152. 10.1515/acve-2017-0013. PMC5798647 29416226

[ref46] Hoornweg T. E.; Bouma E. M.; van de Pol D. P. I.; Rodenhuis-Zybert I. A.; Smit J. M. Chikungunya Virus Requires an Intact Microtubule Network for Efficient Viral Genome Delivery. PLoS Neglected Trop. Dis. 2020, 14 (8), e0008469 10.1371/journal.pntd.0008469. PMC7413472 32764759

[ref47] Diez R.; Diez M. J.; Garcia J. J.; Rodríguez J. M.; Lopez C.; Fernandez N.; Sierra M.; Sahagun A. M. Improvement of Albendazole Bioavailability with Menbutone Administration in Sheep. Animals 2022, 12 (4), 463 10.3390/ani12040463. 35203171 PMC8868263

[ref48] Chen J.; Dai L.; Goldstein A.; Zhang H.; Tang W.; Forrest J. C.; Post S. R.; Chen X.; Qin Z. Identification of New Antiviral Agents against Kaposi’s Sarcoma-Associated Herpesvirus (KSHV) by High-Throughput Drug Screening Reveals the Role of Histamine-Related Signaling in Promoting Viral Lytic Reactivation. PLoS Pathog. 2019, 15 (12), e1008156 10.1371/JOURNAL.PPAT.1008156. 31790497 PMC6907871

[ref49] Rausch K.; Hackett B. A.; Weinbren N. L.; Reeder S. M.; Sadovsky Y.; Hunter C. A.; Schultz D. C.; Coyne C. B.; Cherry S. Screening Bioactives Reveals Nanchangmycin as a Broad Spectrum Antiviral Active against Zika Virus. Cell Rep. 2017, 18 (3), 804–815. 10.1016/j.celrep.2016.12.068. 28099856 PMC5270376

[ref50] Adalja A.; Inglesby T. Broad-Spectrum Antiviral Agents: A Crucial Pandemic Tool. Expert Rev. Anti. Infect. Ther. 2019, 17 (7), 467–470. 10.1080/14787210.2019.1635009. 31216912 PMC7103698

[ref51] Schneidman-Duhovny D.; Dror O.; Inbar Y.; Nussinov R.; Wolfson H. J. PharmaGist: A Webserver for Ligand-Based Pharmacophore Detection. Nucleic Acids Res. 2008, 36, W223–W228. 10.1093/nar/gkn187. 18424800 PMC2447755

[ref52] Sunseri J.; Koes D. R. Pharmit: Interactive Exploration of Chemical Space. Nucleic Acids Res. 2016, 44, W442–W448. 10.1093/nar/gkw287. 27095195 PMC4987880

[ref53] Lagorce D.; Bouslama L.; Becot J.; Miteva M. A.; Villoutreix B. O. FAF-Drugs4: Free ADME-Tox Filtering Computations for Chemical Biology and Early Stages Drug Discovery. Bioinformatics 2017, 33 (22), 3658–3660. 10.1093/bioinformatics/btx491. 28961788

[ref54] Empereur-Mot C.; Zagury J.-F.; Montes M. Screening Explorer-An Interactive Tool for the Analysis of Screening Results. J. Chem. Inf. Model. 2016, 56 (12), 2281–2286. 10.1021/acs.jcim.6b00283. 27808512

[ref55] Levitt N. H.; Ramsburg H. H.; Hasty S. E.; Repik P. M.; Cole F. E.; Lupton H. W. Development of an Attenuated Strain of Chikungunya Virus for Use in Vaccine Production. Vaccine 1986, 4 (3), 157–162. 10.1016/0264-410X(86)90003-4. 3020820

[ref56] Cirne-Santos C. C.; Barros C. d. S.; Nogueira C. C. R.; Azevedo R. C.; Yamamoto K. A.; Meira G. L. S.; Vasconcelos Z. F. M. d.; Ratcliffe N. A.; Teixeira V. L.; Schmidt-Chanasit J.; Ferreira D. F.; Paixão I. C. N. d. P. Inhibition by Marine Algae of Chikungunya Virus Isolated from Patients in a Recent Disease Outbreak in Rio de Janeiro. Front. Microbiol. 2019, 10, 2426. 10.3389/fmicb.2019.02426. 31708898 PMC6821653

[ref57] Khan M.; Santhosh S. R.; Tiwari M.; Lakshmana Rao P. V.; Parida M. Assessment of in Vitro Prophylactic and Therapeutic Efficacy of Chloroquine against Chikungunya Virus in Vero Cells. J. Med. Virol. 2010, 82 (5), 817–824. 10.1002/jmv.21663. 20336760 PMC7166494

[ref58] Rabelo V. W. H.; Da Silva V. D.; Sanchez Nuñez M. L.; Amorim L. d. S. C.; Buarque C. D.; Kuhn R. J.; Abreu P. A.; Paixão I. C. N. D. P. Antiviral Evaluation of 1,4-Disubstituted-1,2,3-Triazole Derivatives against Chikungunya Virus. Future Virol. 2023, 18 (13), 865–880. 10.2217/FVL-2023-0142. 37974899 PMC10636642

