# Supplementary material for: In Silico Drug Repurposing Uncovered the Antiviral Potential of the Antiparasitic Drug Oxibendazole Against the Chikungunya Virus
Source: ACS Omega. 2024 Jun 13;9(25):27632–42. doi: 10.1021/acsomega.4c03417 (PMC11209700; doi:10.1021/acsomega.4c03417)
Supplement: Supplementary file 1 — ao4c03417_si_001.pdf [file ao4c03417_si_001.pdf]

***In silico* drug repurposing uncovered the antiviral potential of the antiparasitic drug oxibendazole against the Chikungunya virus**

Vitor W. Rabelo[a], Maria Leonisa Sanchez-Nuñez[a], Leonardo S. Corrêa-Amorim[a,b], Richard J. Kuhn[c,d], Paula A. Abreu[e], Izabel C. N. P. Paixão[a,f*]

[a]Programa de Pós-graduação em Ciências e Biotecnologia, Instituto de Biologia, Universidade Federal Fluminense, Niterói, RJ, CEP 24210-201, Brazil;
[b]Gerência de Desenvolvimento Tecnológico, Instituto Vital Brazil, Niterói, RJ, 24230-410, Brazil;
[c]Department of Biological Sciences, Purdue University, West Lafayette, IN 47907, USA;
[d]Purdue Institute of Inflammation, Immunology, and Infectious Disease, Purdue University, West Lafayette, IN 47907, USA;
[e]Instituto de Biodiversidade e Sustentabilidade (NUPEM), Universidade Federal do Rio de Janeiro - Campus Macaé, RJ, CEP 27965-045, Brazil;
[f]Departamento de Biologia Celular e Molecular, Instituto de Biologia, Universidade Federal Fluminense, Niterói, RJ, CEP 24210-201, Brazil.

*Email: izabelpaixao@id.uff.br

CONTENTS

Supplementary Figure S1. Pharmacophore models developed based on known CHIKV nsP2 protease inhibitors.

Supplementary Figure S2. Cytotoxic and antiviral effects of oxibendazole (OBZ) and the control drug chloroquine (CQ) against the replication of CHIKV BRA/RJ/18 strain in Vero cells.

Supplementary Table S1. Recovery rates of known CHIKV nsP2 protease inhibitors calculated for the developed pharmacophore models. For validation studies, all inhibitors were included, except the ones used in the model construction.

Supplementary Table S2. Area under the ROC curve (AUC-ROC) values calculated for a set of known CHIKV nsP2 protease inhibitors and decoys, using different isolated or combined scoring functions.

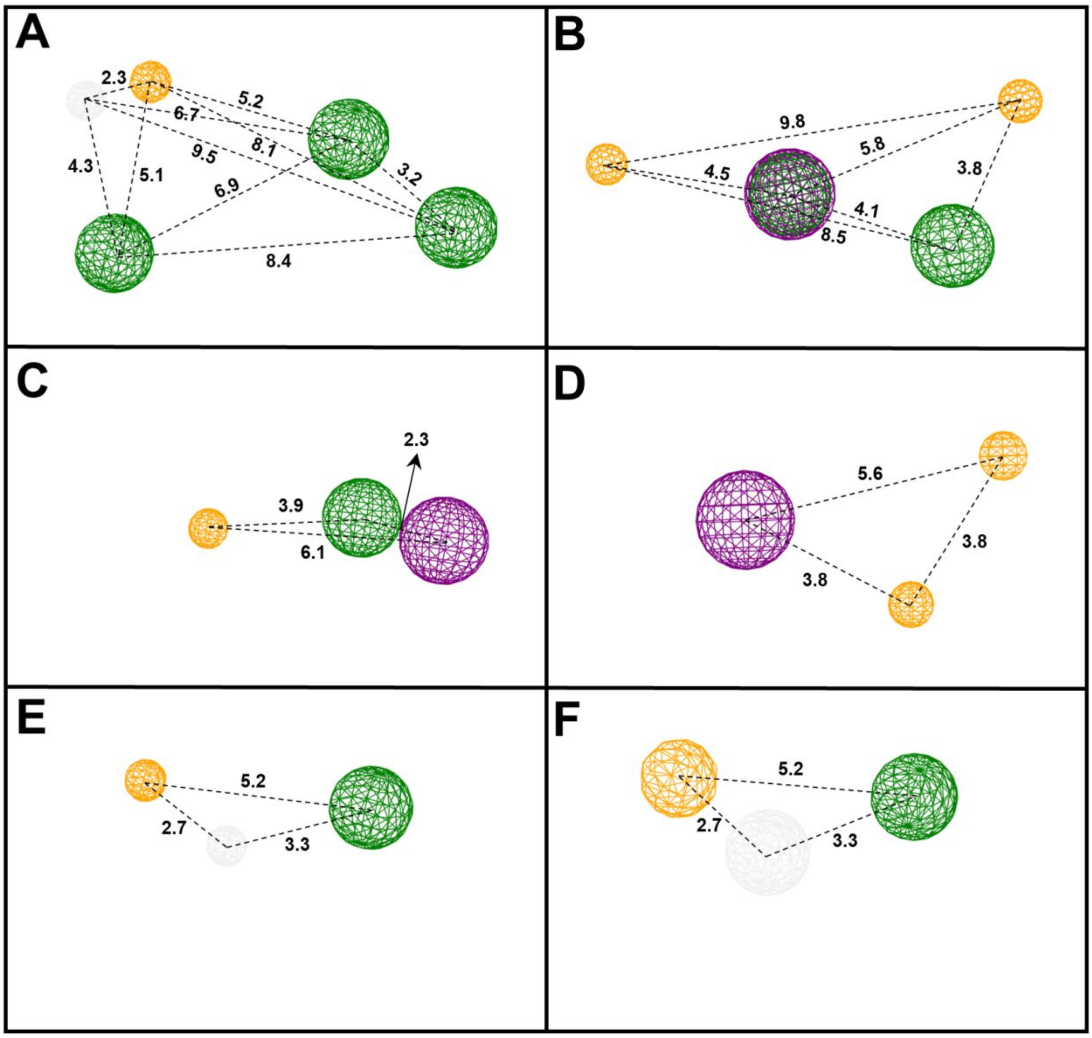

Supplementary Figure S1. Pharmacophore models developed based on known CHIKV nsP2 protease inhibitors. Models were manually constructed using the conformation of (A) (*RRS*)-**C5**, and (**B**) **C12**; or models were constructed using the Pharmagist server, based on the inhibitors (**C**) (*RRS*)-**C5**, (*S*)-**C9** and **C12**, (**D**) (*SS*)-**C7**, (*S*)-**C9** and **C12**; and (*RRS*)-**C5**, (*SS*)-**C7** and (*S*)-**C9** with radius values of (**E**) 0.5 and 0.5 Å or (**F**) 0.9 and 1.0 Å for the hydrogen bond (H-bond) acceptor and donors, respectively. H-bond acceptor and donor groups are shown in orange and gray, respectively, while hydrophobic and aromatic groups are represented in green and purple, respectively. For the generation of these models, the binding mode of the inhibitors with the protein reported previously[1] was used. Distances between the pharmacophore groups are also shown (in Å).

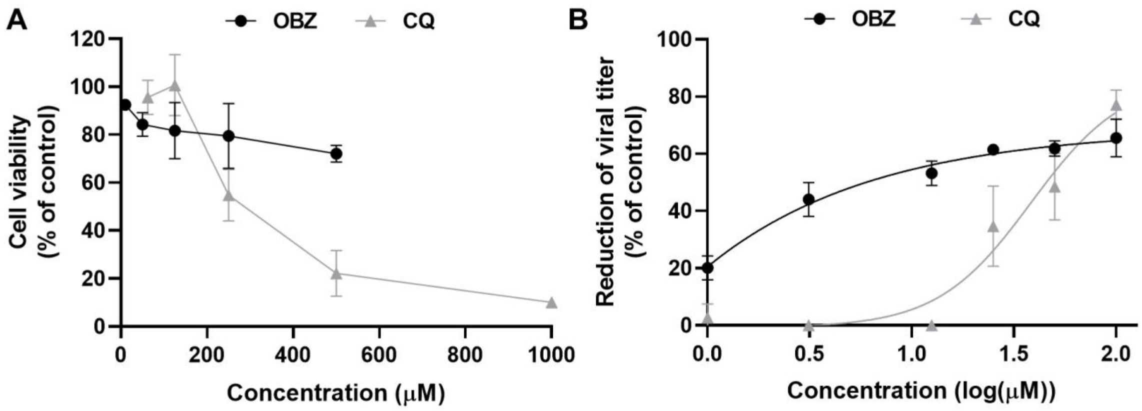

Supplementary Figure S2. Cytotoxic and antiviral effects of oxibendazole (OBZ) and the control drug chloroquine (CQ) against the replication of CHIKV BRA/RJ/18 strain in Vero cells. (A) Cytotoxicity of the compounds was evaluated by MTT assay after treatment of Vero cells (2 x 10^4 cells/well) for 72h. (B) Vero cells were infected with CHIKV (MOI 1) for 2h, and then, cells were treated with CQ or OBZ at different concentrations (1.0, 3.125, 12.5, 25, 50, and 100 μM) for 24h. At 24 hpi, cells were lysed by three cycles of freezing and thawing, the supernatants were harvested, and virus titers were determined by plaque assays. Rates of the reduction of viral titer (%) were calculated related to infected and untreated controls. Data are expressed as mean ± standard deviation of three independent experiments measured in triplicate.

Supplementary Table S1. Recovery rates of known CHIKV nsP2 protease inhibitors calculated for the developed pharmacophore models. For validation studies, all inhibitors were included, except the ones used in the model construction.

**Pharmacophore model**	**Recovered inhibitors**		
	**N_{total}**	**$N_{recovered}$**	**Recovery rate (%)**
A	13	2	15.38
B	13	0	0.00
C	12	5	41.67
D	12	6	50.00
E	12	9	75.00
F	12	12	100.00

Supplementary Table S2. Area under the ROC curve (AUC-ROC) values calculated for a set of known CHIKV nsP2 protease inhibitors and decoys, using different isolated or combined scoring functions. For consensus scoring function analysis, the combinations that resulted in AUC-ROC values ≥ 0.7 are shown.

**Scoring function**	**AUC-ROC**
Autodock 4.2.6	0.65
Autodock Vina	0.66
smina	0.67
vinardo	0.62
Chemscore	0.52
ASP	0.58
ChemPLP	0.57
Goldscore	0.52
ad4_scoring	0.71
DSX	0.65
smina + ad4_scoring	0.71
smina + vinardo	0.70
smina + vinardo + ad4_scoring	0.72
smina + vinardo + ad4_scoring + DSX	0.70

References

(1) Rabelo, V. W.-H.; Paixão, I. C. N. de P.; Abreu, P. A. Structural Insights into the Inhibition of the NsP2 Protease from Chikungunya Virus by Molecular Modeling Approaches. *J. Mol. Model.* **2022**, *28* (10), 311. https://doi.org/10.1007/S00894-022-05316-3.
